# Prophetic Granger Causality to infer gene regulatory networks

**DOI:** 10.1371/journal.pone.0170340

**Published:** 2017-12-06

**Authors:** Daniel E. Carlin, Evan O. Paull, Kiley Graim, Christopher K. Wong, Adrian Bivol, Peter Ryabinin, Kyle Ellrott, Artem Sokolov, Joshua M. Stuart

**Affiliations:** 1 University of California San Diego, Department of Medicine, La Jolla, CA, United States of America; 2 University of California Santa Cruz, Department of Biomolecular Engineering, Santa Cruz, CA, United States of America; 3 Oregon Health Sciences University, Department of Biomedical Engineering, Portland, OR, United States of America; Tampere University of Technology, FINLAND

## Abstract

We introduce a novel method called Prophetic Granger Causality (PGC) for inferring gene regulatory networks (GRNs) from protein-level time series data. The method uses an L1-penalized regression adaptation of Granger Causality to model protein levels as a function of time, stimuli, and other perturbations. When combined with a data-independent network prior, the framework outperformed all other methods submitted to the HPN-DREAM 8 breast cancer network inference challenge. Our investigations reveal that PGC provides complementary information to other approaches, raising the performance of ensemble learners, while on its own achieves moderate performance. Thus, PGC serves as a valuable new tool in the bioinformatics toolkit for analyzing temporal datasets. We investigate the general and cell-specific interactions predicted by our method and find several novel interactions, demonstrating the utility of the approach in charting new tumor wiring.

## Introduction

A major goal of systems biology is to infer the genetic “circuitry” that governs how cells respond to environmental stimuli, developmental cues, and therapeutic interventions. The challenge is to find a gene regulatory network (GRN) that can accurately predict the consequences of *perturbations* not seen during model construction. We use the term GRN loosely to describe any set of *directed* influences between genes and gene protein products. This encompasses transcriptional regulatory networks that describe transcription factors acting alone or in complexes to affect the mRNA production of target genes through activation of their *cis-*regulatory elements. It also includes protein-protein signaling modifications such as phosphorylation and ubiquitination that are either signal transducing or amplified by hierarchical cascades of modifiers (e.g. MAP-kinases). While the time-scales of the transcriptional and post-transcriptional interacting relationships cover a wide range, they all describe cause-effect relations between genes and the products encoded by them. Reverse-engineering this wiring from high-throughput datasets remains a difficult problem.

Nearly two decades of research in systems biology has introduced many approaches for inferring GRNs from data. Some of the first approaches inferred genetic relationships from steady state datasets using correlation (e.g. observed in multiple species [1]) or information-theoretic measures of dependence (e.g. Relevance Networks [2]). Later approaches provided insightful advances including Weighted Gene Correlation Analysis to generalize network topology analyses to use continuous measures of similarity [3], ARACNE [4] to eliminate redundant connections, Context Likelihood of Relatedness [5] to enrich for direct interactions using a Z-transform on mutual information distributions, and Bayesian Networks [6] to infer consistent probabilistic dependencies that formalize the previous mutual information approaches to name a few. Machine-learning methods have also had success, exemplified by the decision tree approach of the GENIE3 method [7] that has performed well in multiple benchmarks. A recent review and comparison of methods for inferring GRNs from steady state data can be found in [8,9].

Perturbation and time series data provide key information for inferring causation, enabling the directionality of protein-to-protein influence to be identified. Several methods for dynamic modeling have been introduced to capitalize on the growing availability of such data including Boolean Networks [10], Dynamic Bayesian Networks [11,12], Factor Graphs to capture Nested Effects [13], and Granger Causality [14–16]. For a recent comparison of dynamic models see [17] as well as the results of recent DREAM challenges [18–20].

One difficulty in the field of GRN inference is the ability to unequivocally evaluate methods as gold standard datasets are in limited supply. The DREAM series of challenges was launched to formalize the creation of benchmarks. While the choice of metric for DREAM challenges may be somewhat arbitrary with several possible alternatives available, they have the distinct advantage of eliminating the so called “self assessment trap” in which method’s developer’s either consciously or unconsciously bias the evaluation in favor of their own methods [21].

DREAM has often found that ‘wisdom of crowds’ approaches combining several strategies often perform better than any stand-alone approach [22], consistent with classic work on ensembles that demonstrate weak learners can be combined to form a more accurate method as the errors of the weak learners tend to be mutually uncorrelated and average out [23,24]. The accuracy of the top-performing ensembles reveal that considerable room for improvement exists in the ability of individual methods to reverse-engineer GRNs. New methodology, or those that draw inspiration from different fields of research, could complement existing algorithms.

In this paper, we describe a novel Prophetic Granger Causality (PGC) approach for inferring a GRN from time series data. The method introduces a regularized regression framework inspired by Granger Causality [25] that appropriately handles irregularly spaced time intervals. In contrast to the traditional Granger Causality approach that uses only past observations, we introduce a “prophetic” extension that also includes future observations, to consider interaction evidence from the perspective of both the regulator and the target.

The PGC method, when augmented with the prior, was found to be more accurate than 73 other methods submitted to the Health Provider Network (HPN) 1A sub-challenge [26]. Contestants were given a time series of phosphoproteomics data on several breast cancer cell lines following ligand stimulation and, for each cell line, asked to infer a directed protein-protein signaling network. In this paper, we investigate PGC’s usefulness in providing novel predictive information as part of an ensemble. We also demonstrate that the “prophetic” extension benefits not only PGC but also improves on a method called GENIE3, a winning approach in previous DREAM network inference challenges. Finally, results on a yeast time series dataset indicates the approach will generalize to other GRN inference problems.

## Methods

### Network inference with Prophetic Granger Causality

**Fig 1:**
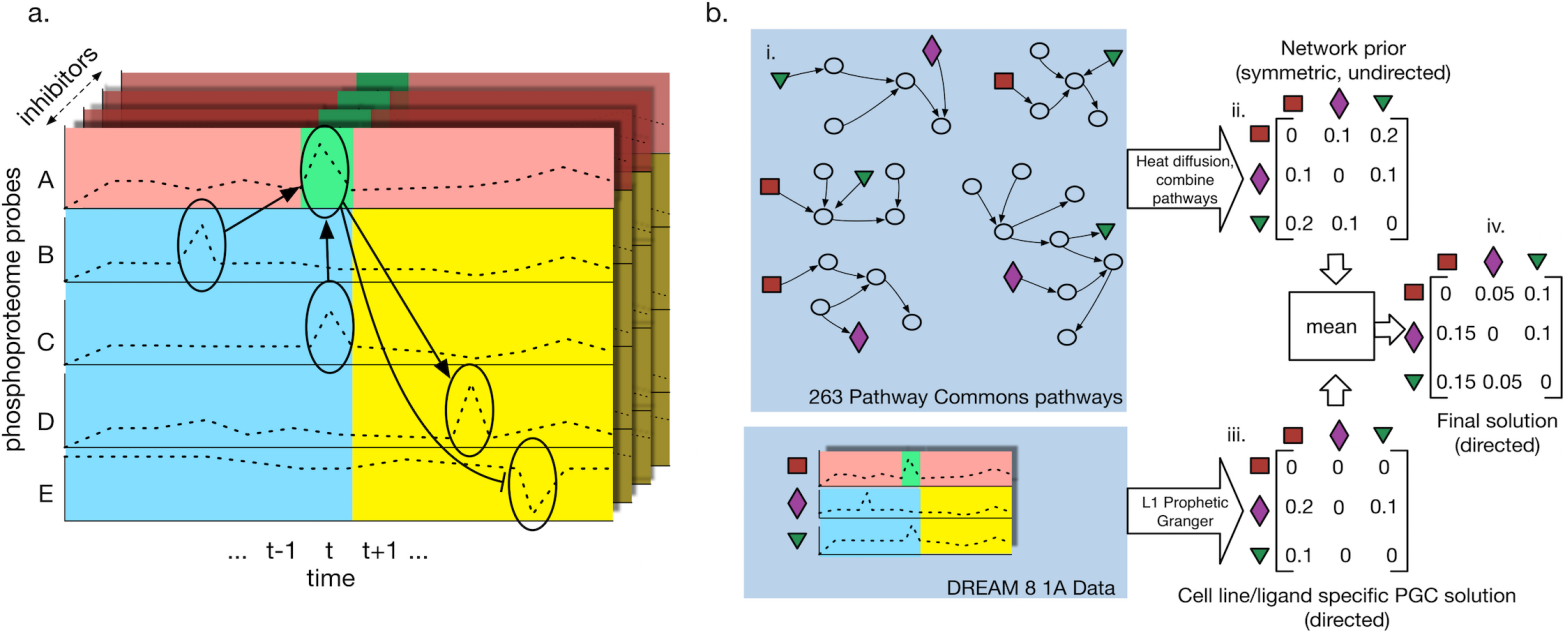
Prophetic Granger Causality method. (A) The method is given a set of probes (rows; y-axis) measuring the level of a particular phospho-protein state at particular time points (columns; x-axis). Each probe value at each time point) is considered in turn as a linear regression of all other feature times and probes. Depicted is probe *A* being considered at time *t* (green). The penalty parameter L1 is chosen such that autoregression contributions (red) are set to zero. Any remaining non-zero regression coefficients for other probes suggest causality; past or concurrent time point probes (blue) are considered *causal of* the target; future time point probes (yellow) are considered to be *caused by* the target. The different inhibitor conditions are treated as different examples in the regression task. This process was repeated for each time and probe, with each regression task contributing to the final connectivity matrix. (B) Overview of the overall PGC plus network prior approach for the HPN DREAM8 submission. Shown is a prediction for a single (cell line, ligand) pair task. (i.) 263 Pathway Commons pathways having at least two proteins in the DREAM dataset (colored shapes). (ii.) Heat diffusion kernel used to measure closeness between protein pairs in each pathway (see S1 File) were combined into a single weighted “network prior,” represented as an adjacency matrix. (iii.) The Prophetic Granger solution, obtained as shown in part A. (iv.) The final solution for the (cell line, ligand stimulus)-pair is produced by averaging the network prior with the absolute value of the Prophetic Granger solution.

As input we are given a dataset *X* containing a collection of *n* separate time series, each probing the levels of a set of *m* proteins (see Fig 1A for an example with the HPN DREAM challenge data). We view *X* as a matrix where each of the n*m entries, *x*_*i*,*p*_, is a time series replicate for protein *p*, each containing *T* observed levels across the time points *t = 1…T*. Entry *x*_*i*,*p*,*t*_ represents the level of protein *p* in time series *i* at time point *t*. The time series are assumed to be in register with each other such that the same proteins are measured at the same corresponding time intervals. This allows the use of a fixed but arbitrary ordering over the protein-time pairs. Denote such an ordering of pairs as the vector ***z*** and let *π(z*_*j*_*)* represent the *j*^th^ protein and *τ(z*_*j*_*)* the *j*^th^ time point contained in the *j*th pair.

The Granger approach searches for explanatory states in the past that best predict observed levels in the present. We consider predicting *x*_*i*,*p*,*t*_ from the other observed levels and rewrite *x*_*i*,*p*,*t*_ as *y*_*i*,*p*,*t*_ to indicate its use as the response variable in the regression formulations described next. We begin with the LASSO-Granger method [27] in which the predicted level for *y*_*i*,*p*,*t*_ is a linear combination of the past and present:

$${\hat{y}}_{i,p,t}=\sum_{u<t}\alpha_{u}x_{i,p,u}+\sum_{\begin{array}{c} j:\tau (z_{j})\leq t,\\ \pi (z_{j})\neq p \end{array}}\beta_{j}x_{i,\pi (z_{j}),\tau (z_{j})}+\beta_{0}$$ (Eq 1)

where the vector *α* contains coefficients for the *autoregression* terms–the past states of protein *p* represented in the first term of the summation–and *β* contains coefficients for the *exogenous* terms–past and present states of all other proteins.

We introduce a *prophetic* extension to the above formulation to include future states. In this situation, the regression is allowed to find target changes in the future that are predictive of a regulator’s state in the past. Regressing in the usual forward-time direction, in which a target *p* is used as the response variable, may miss detecting the influence of a particular regulator *q* when *p* has many regulators. This may happen because the other regulators provide enough explanatory power to predict *p*’s state, making *q*’s information redundant (see Supplemental S1 Fig). The intuition of the prophetic extension is that *p*’s state in the future could provide (even partial) predictive power for *q*’s state in the past. We rewrite the regression to obtain the prophetic extension:

$${\hat{y}}_{i,p,t}=\sum_{u<t}\alpha_{u}x_{i,p,u}+\sum_{j:\pi (z_{j})\neq p}\beta_{j}x_{i,\pi (z_{j}),\tau (z_{j})}+\beta_{0}$$ (Eq 2)

where the only difference between the above and Eq (1) is the inclusion of future state levels represented in the time point selections of the summations.

We now turn to the task of solving for an optimal setting of the coefficients. A regularization strategy selects for sparse models with few non-zero valued coefficients. In our approach, we use the squared error loss combined with a LASSO regularization penalty:

$${\text{min}}_{\alpha ,\beta }\left\{\frac{1}{2}\sum_{i=1}^{n}{\left(y_{i,p,t}-{\hat{y}}_{i,p,t}\right)}^{2}+\lambda (\sum_{u\neq t}|\alpha_{u}|+\sum_{j:\pi (z_{j})\neq p}\left|\beta_{j}\right|)\right\}$$ (Eq 3)

where *y*_*i*,*p*,*t*_ is the observed level (i.e. equal to *x*_*i*,*p*,*t*_) and ${\hat{y}}_{i,p,t}$ is the estimated level given in Eq (2). Note that all levels of *p* across all *n* time series are included. The *λ* parameter determines the strength of the regularization and the sparsity of the regression coefficients. The regression problem can be solved using coordinate descent [28], a standard optimization method for solving regression problems with a LASSO penalty term. It works particularly well because coefficients that get “snapped” to zero by the softmax operator (see Eqs (4) and (5) below) will often remain at zero and require no further updates, which leads to efficient runtimes [29]. Specifically, the following update rule provides a new estimate for the *u*^th^ (*u≠t*) autoregression term:

$$\alpha_{u}\leftarrow \frac{S(\sum_{i=1}^{n}x_{i,p,u}({\hat{y}}_{i,p,t}-{\hat{y}}_{i,p,t}^{\left(-u\right)}),\lambda )}{\sum_{i=1}^{n}x_{i,p,u}^{2}}$$ (Eq 4)

where ${\hat{y}}_{i,p,t}^{\left(-u\right)}=\sum_{v\neq t,u}\alpha_{v}x_{i,p,v}+\sum_{j:\tau (z_{j})\neq t,u}\beta_{j}x_{i,\pi (z_{j}),\tau (z_{j})}+\beta_{0}$ is a model that excludes terms from time point *u*. Note that exogenous terms need not be included in u, since α is an autoregression term (i.e., weight for features that encode information from the same time series but other time points). The update in Eq (4) is based on the difference between a model that contains, and one that lacks, information from time point *u*. *S(a*,*b)* is a soft-threshold operator that eliminates terms with contributions deemed too small by “snapping” its first argument to zero when the absolute value falls below the value of the second argument [30]; i.e. *S*(*a,b*) = sign(a)(|*a*| − |*b*|)_+_.

An analogous update rule can be used for the *β* weights. If *q* is the *j*^th^ protein (i.e. *q = π(z*_*j*_*)*) and *u* is the *j*^th^ time point (i.e. *u = τ(z*_*j*_*)*), then the *j*^th^ exogenous coefficient can be updated using the rule:

$$\beta_{j}\leftarrow \frac{S\;(\sum_{i=1}^{n}x_{i,q,u}({\hat{y}}_{i,p,t}-{\hat{y}}_{i,p,t}^{\left(-(q,u)\right)}),\lambda )}{\sum_{i=1}^{n}x_{i,q,u}^{2}}$$ (Eq 5)

and ${\hat{y}}_{i,p,t}^{\left(-(q,u)\right)}=\sum_{v\neq t}\alpha_{v}x_{i,p,v}+\sum_{\begin{array}{c} j:\tau (z_{j})\neq t,u,\\ \pi (z_{j})\neq p,q \end{array}}\beta_{j}x_{i,\pi (z_{j}),\tau (z_{j})}+\beta_{0}$ is the model without the inclusion of the *j*^th^ protein-time pair from the exogenous terms.

The meta-parameter *λ* controls the sparsity of the resulting solution. Larger values result in higher numbers of eliminated coefficients. The key Granger-inspired step is to set *λ* so that all autoregression terms are zeroed out. This is consistent with the classical Granger approach that measures the predictive power gained from another time series over simple auto-regression [2]. We follow this intuition by rearranging Eq (4) to obtain the upper bound on *λ*_*0*_ where all the autoregression terms are zero:

$$\lambda_{0}={\text{max}}_{u}\left|\frac{1}{n}\sum_{i=1}^{n}x_{i,p,u}({\hat{y}}_{i,p,t}-{\hat{y}}_{i,p,t}^{\left(-u\right)})\right|$$ (Eq 6)

One can verify that *λ*_*0*_ becomes the second argument in the soft-threshold operator of Eq (4) when all of the weights in *α* are set to zero. Using this setting and solving the regression problem in Eq (3) results in a solution where all of the autoregression terms are ignored and any remaining predictors are contributed by exogenous terms recorded in *β*, which are interpreted as evidence of causal relationships. Of course, it is possible that setting *λ = λ*_*0*_ also causes all *β* coefficients to vanish as well. Such cases are interpreted as a lack of evidence for causality for *p*.

### Construction of the predicted network in a connectivity matrix

We estimate the importance of protein *q* in predicting *p*’s levels in time series *i* by aggregating all of its non-zero contributions recorded in the weight vector *β*. We accumulate causal information across all regression tasks in a matrix *C*, where entry *C*_*q*,*p*_ represents the directed prediction that *q*’s state exerts a causal influence on *p*’s state. Before any regressions are performed, *C* is initialized to the matrix of all zeros. Then, after regressing on protein *p*, the following update rule is executed for every possible predictor *q≠p*, extracting two types of causal evidence from *β* recorded in *C*:

$$\forall_{j:\pi (z_{j})=q}\left\{ \begin{array}{c} C_{q,p}\leftarrow C_{q,p}+\frac{\beta_{j}}{\sum_{k}\left|\beta_{k}\right|},\tau (z_{j})\leq t\\ C_{p,q}\leftarrow C_{p,q}+\frac{\beta_{j}}{\sum_{k}\left|\beta_{k}\right|},\tau (z_{j})>t \end{array}\right.$$ (Eq 7)

In the top case, *q* is predictive of *p*’s future state; i.e., *q* has some non-zero entries in *β* with associated time points before or concomitant with *t*. This is the usual Granger causality situation. On the other hand, the prophetic update on the bottom occurs if a predictor variable’s state occurs in the *future*; i.e. *q*’s non-zero *β* entries occur *after time t*. In this case, the matrix records that *p* may be a causal influence of *q* (see bottom part of Eq (7)). Note that the directionality of causality updated in this step may not match the predictor→target directionality of the regression. In this way, a final set of directed protein → protein interactions are collected in *C* after all proteins and all of their time points are considered as regression targets in turn.

### Network inference with GENIE3

The “prophetic” concept can be used in conjunction with other regression models including non-linear variants. To test its merits in an additional setting, we explored its use as an addendum to GENIE3 [31], a method that won the DREAM5 network inference challenge and has been shown to be effective for inferring biological networks from expression data [22]. We briefly describe here a prophetic Granger extension to GENIE3.

Eq (3) is a linear variant of the more general regression problem:

$${\hat{y}}_{i,p,t}=f\;\left(x_{i}^{\left(-(p,t)\right)}\right)$$ (Eq 8)

where $x_{i}^{\left(-(p,t)\right)}$ represent all of the data points excluding the particular protein *p* at time point *t* that is the target of the regression. GENIE3 uses a random-forest classifier as the function *f* and sets its parameters to minimize the squared error loss $\sum_{i=1}^{n}{\left(y_{i,p,t}-{\hat{y}}_{i,p,t}\right)}^{2}$. A tree defines a recursive nesting of training sample splits according to a set of binary tests, represented as decision nodes. Each decision node uses either an autoregressive or exogenous variable from $x_{i}^{\left(-(p,t)\right)}$, as the binary split, chosen to reduce the variance of the time points in *y*_*i*,*p*,*t*_ remaining to be classified under the context of the current sub-tree. The selection of a term at a higher level in a tree than another is evidence the first has more predictive information than the second when both are used on their own.

Through bootstraps of the data, GENIE3 produces different random forests for the regression task. The importance of a predictor variable can then be estimated from the amount of variance it splits each time it is used as a decision node across bootstrap replicates and across all of the trees it is used in.

As in the Granger regression case, Genie3 produces an estimation of the importance of every element of $x_{i}^{\left(-(p,t)\right)}$ in predicting *y*_*i*,*p*,*t*_ called β^GENIE3^. Where in the PGC case the terms in β associated with the autoregressive terms were zero by construction of the algorithm, in this case we set them to zero so that they don’t contribute to C^GENIE3^. Once calculated, *β*^*GENIE3*^ is then used in place of *β* in Eq (7) to derive GENIE3’s own causality matrix *C*^*GENIE3*^ across all regression tasks.

### Adding a network prior to the predicted GRN

Rather than use the inferred GRNs from regression methods alone, we tested their performance when their predictions were added to a *network prior*. The network prior was computed using only interactions found in the literature and without regard to the time series dataset. A heat diffusion approach was used to find interactions among the set of proteins using a pathway interaction database (see S1 File). The resulting network is an undirected Gene Interaction Network (GIN), recorded in the symmetric matrix *B*.

The final matrix *F*, which describes the directed GRN, is obtained by combining the undirected network prior *B* and *C*, an assymetric matrix encoding the causal relations inferred by PGC. To facilitate combining the matrices (Fig 1B), all of the entries in the strictly positive matrix *B* and *C* were scaled to the interval [0,1] by dividing by the largest entry in each matrix. *F* was then computed by taking the arithmetic mean:

$$F=\frac{1}{2}\left(\frac{{\left|C\right|}_{*,*}}{\max({\left|C\right|}_{*,*}}+\frac{B}{\max(B)}\right)$$ (Eq 9)

where |C|_*,*_ returns a new matrix containing the element-wise absolute values of the matrix C. Note that averaging the networks together has the effect of “orienting” some of the edges in the undirected GIN defined by *B* using the weightings in the GRN *C*. This produces an overall directed GRN because the result is a non-symmetric matrix of interactions recorded in *F*. Other combinations of *C* and *B* are possible and were explored in the community-participation stage of the challenge [26], but the simple averaging scheme performed well enough to take the top-performing position in the challenge. Other weighting schemes were also explored (S2 Fig), and while we observe a slight (3%) improvement on the simple averaging scheme, we find that weightings between 50% and 90% are fairly comparable, with the 80% weighting on the prior achieving the highest level of performance.

PCG and Prophetic GENIE3 code can be found at https://github.com/decarlin/prophetic-granger-causality.git

## Results

### Description of the HPN DREAM 8 data set

The Heritage Provider Network DREAM 8 Breast Cancer Network Prediction Challenge was a contest to predict causal protein networks from time series reverse phase protein array (RPPA) data. The *in vitro* portion of the challenge provided 4 cell lines (BT549, BT20, MCF7, and UACC812) observed in the presence of 4 inhibitor conditions (AKT, AKT + MET, FGFR1 + FGFR3, and DMSO control) exposed to one of 8 ligand stimuli (Serum, PSB, EGF, Insulin, FGF1, HGF, NRG1, and IGF1). The RPPA data was taken at time points t = 0, 5 min, 15 min, 30 min, 1 hr, 2 hr, and 4 hr. From this data, challenge participants were asked to produce a network for each (stimulus ligand, cell line) pair, resulting in a total of 32 networks.

The HPN dataset is indexed by five variables including time *t*, cell line *l*, stimulus ligand condition *c*, inhibitor *i*, and phosphoprotein antibody probe *p*. We solve for a context-specific network for each (stimulus ligand, cell line) pair. Therefore, the regression problems are set up by setting the time-series matrix *X* for a (stimulus ligand, cell line) pair such that the inhibitors are treated as the replicates. Probes represent the protein levels in *X*; any proteins with multiple probes are first averaged together in *X* (see Fig 1A).

The contest organizers evaluated 73 different methods for this challenge. One inhibitor was withheld from participants. Targets were identified as those proteins that had a significant change in activity upon inhibition with the withheld agent. In this way, targets for each withheld protein were determined for each (stimulus ligand, cell line) pair. A network submitted by a challenge participant was evaluated by counting the number of predicted downstream relations in common with the experimental results. Sweeping through a prediction score threshold created an area under the receiver operator curve (AUROC) for each predicted network. The average AUROC of all 32 networks was the final scoring metric used in the challenge.

We report average AUROC alone for discussing results that pertain to the HPN-DREAM8 challenge. For non-HPN-related experiments, we also report area under the Precision-Recall (AUPRC) as an additional metric. AUPRC is better-suited for datasets with a large discrepancy between the numbers of positive and negative examples [32].

### Prophetic Granger solution to the HPN challenge

The Methods section describes the top-performing PGC approach submitted to the HPN DREAM8 1A sub-challenge. In that submission, each (stimulus ligand, cell line) pair was treated as a separate regression task. Since the evaluation criteria of the challenge made no distinction between excitatory and inhibitory links, the absolute value of the connectivity matrix *C* was used in order to consider both types of causal interactions. The AUROC achieved by this approach after its combination with the network prior was 0.785, which was only a marginal improvement over the prior alone (average AUROC = 0.783). For reference, the next best method was contributed by a different team and achieved an average AUROC of 0.755. The method was based on a time-lagged linear correlation method that refined its predictions against a prior based on the KEGG pathway database [26]. Indeed all of the top-scoring methods used some form of prior, reinforcing the benefit of using biological knowledge in this challenge.

**Fig 2:**
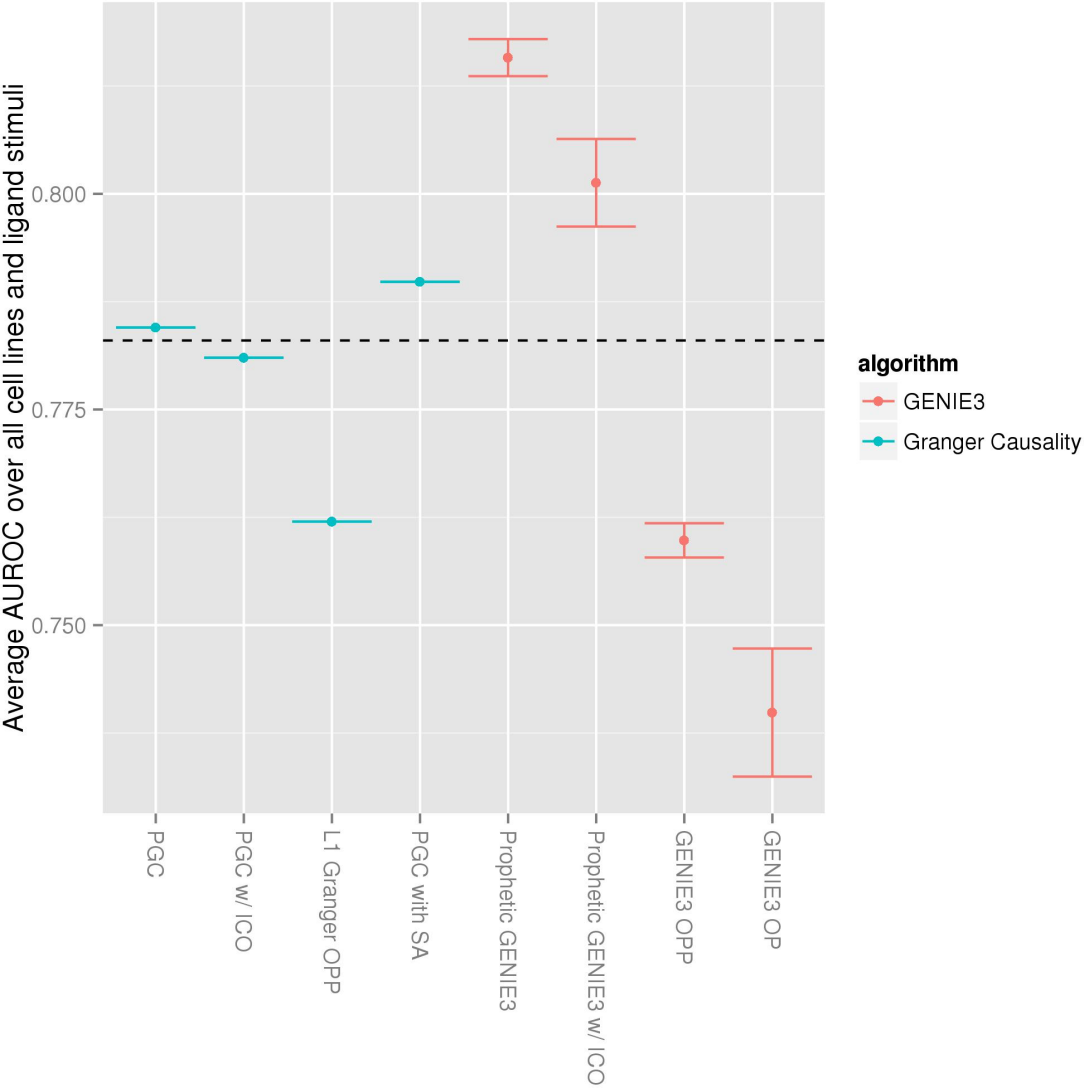
Prophetic augmentations of Granger Causality and GENIE3 complement prior network knowledge. Performance on the HPN DREAM8 1A sub-challenge after combining different methods with the network prior is shown. Performance of the prior alone is represented by the dotted line. Prophetic Granger Causality, PGC; ignorant of causal ordering, ICO; solutions averaged across all experiments, SA; only past and present time points used, OPP (since this regression framework does not use future points, it cannot be called prophetic); only present time points used, OP. GENIE3 OP is the originally published version of the algorithm; since there are not external time points used for this calculation, there is no equivalent Granger algorithm. GENIE3 error bars show one standard deviation of performance with 10 different random seeds.

In the post-challenge analysis, we discovered that further improvements were possible. In particular, defining the regression tasks on a per-cell-line basis in which all 8 ligand stimuli were used together, providing 32 training examples (8 ligand stimuli across 4 inhibitors), gave a higher performance. Combining the resulting cell-line-specific GRNs with the network prior, followed by an averaging into a single *consensus network* yielded an average AUROC of 0.790 (see “solutions averaged across all experiments (PGC with SA)”, Fig 2). The improvement in accuracy suggests that there is little biological variance across the different stimulus ligands, allowing the regression models to make good use of the eight-fold increase in the sample size.

### ‘Prophetic’ use of past and future time points improves network inference

We asked whether the prophetic component of the Granger regression described above provided an advance over the regression alone, as well as whether similar prophetic augmentation could improve an already extant algorithm, GENIE3. We considered both Granger regression and GENIE3 in the context of the DREAM8 1A sub-challenge data to quantify the differences in performance after each method was combined with the same network prior. For both methods, we found that considering future time points helped boost their performance for predicting causality (Fig 2). Also, in both PGC and Prophetic GENIE3, failing to reverse the directionality of an explanatory variable that occurred after the response variable (labeled “PGC w/ ICO” and Prophetic Genie3 w/ ICO” where ICO indicates “ignorant of causal ordering” in Fig 2) lowered performance. This suggests that the relative temporal position of observations does indeed provide information about the causal relationships between the proteins.

Prophetic GENIE3 obtained higher performance than PGC when using all available time-points (past and future) for each regression task. As with PGC, combining the data across stimulus ligands yielded higher accuracy (average AUROC = 0.696, data not shown) than formulating a separate regression task for each (stimulus ligand, cell line) pair (average AUROC = 0.552, data not shown). Furthermore, considering all of the data (i.e. from all cell line and stimuli) at once to produce a single network gave the best performance of Prophetic GENIE3 (average AUROC = 0.722, when combined with the network prior, average AUROC = 0.815) This is the configuration used for the analysis in Fig 2. This result provides further evidence that, when data is scarce, exploiting the full dataset outweighs the possible advantage of fitting specific nuances present in individual cell lines and stimulus ligands.

### PGC provides complementary predictive power for ensemble learning

PGC outperformed several machine learning methods when used in conjunction with the biological prior network (see S3 Fig). Despite this fact, PGC on its own, without the addition of the network prior, achieved mediocre performance on the HPN DREAM8 1A sub-challenge (average AUROC = 0.55). This conundrum suggests that PGC’s errors were appreciable but compensated by the biological prior more readily than errors from other approaches. The information gleaned by PGC might then be orthogonal to other approaches and worth incorporating at some level with ensemble approaches. In an ensemble setting, several weak learners can be highly accurate when used together, if their errors are uncorrelated [33].

Because the findings based on a single dataset could be anecdotal, we further measured PGC’s network inference ability beyond its application to the HPN challenge. To do so, we compared it to other leading network inference methods in the absence of prior knowledge, using data from multiple studies. The methods we considered included the following: EBDBnet [34], which is a dynamic Bayes net approach; Context Likelihood of Relatedness (CLR) [35], which computes a symmetrically normalized mutual information measure that helps enforce relation specificity; ARACNE [36], which is another mutual information approach that accounts for indirect interactions; and ScanBMA [37], a Bayesian method that averages bootstrapped linear regression models using their posterior probabilities. In the case of ScanBMA, we report the results both with and without the prior provided by the authors of the method. The so-called “g-prior” of ScanBMA allows interactions with external support to have higher variance in the associated regression coefficients.

We applied the above methods to the yeast mRNA time series data provided by Yeung *et al*. [38] and to the synthetic data provided by the DREAM4 challenge [39,40]. The Yeung data is a regular mRNA time series generated at ten-minute intervals for 95 genetically diverse yeast strains exposed to rapamycin. The DREAM4 dataset is a simulated dataset created with the GeneNetWeaver software. It simulated regular transcriptional time series for five subnetworks of a gold standard network with ten genes each over 21 time points. The results for the individual methods appear in S1 Table. On these datasets, PGC achieved lower accuracy relative to other approaches without prior knowledge. The GENIE3 method ranked the best on average, although it did not perform as well in the HPN DREAM8. We found that no ensembles of other methods improved on the performance of GENIE3 (data not shown).

**Fig 3:**
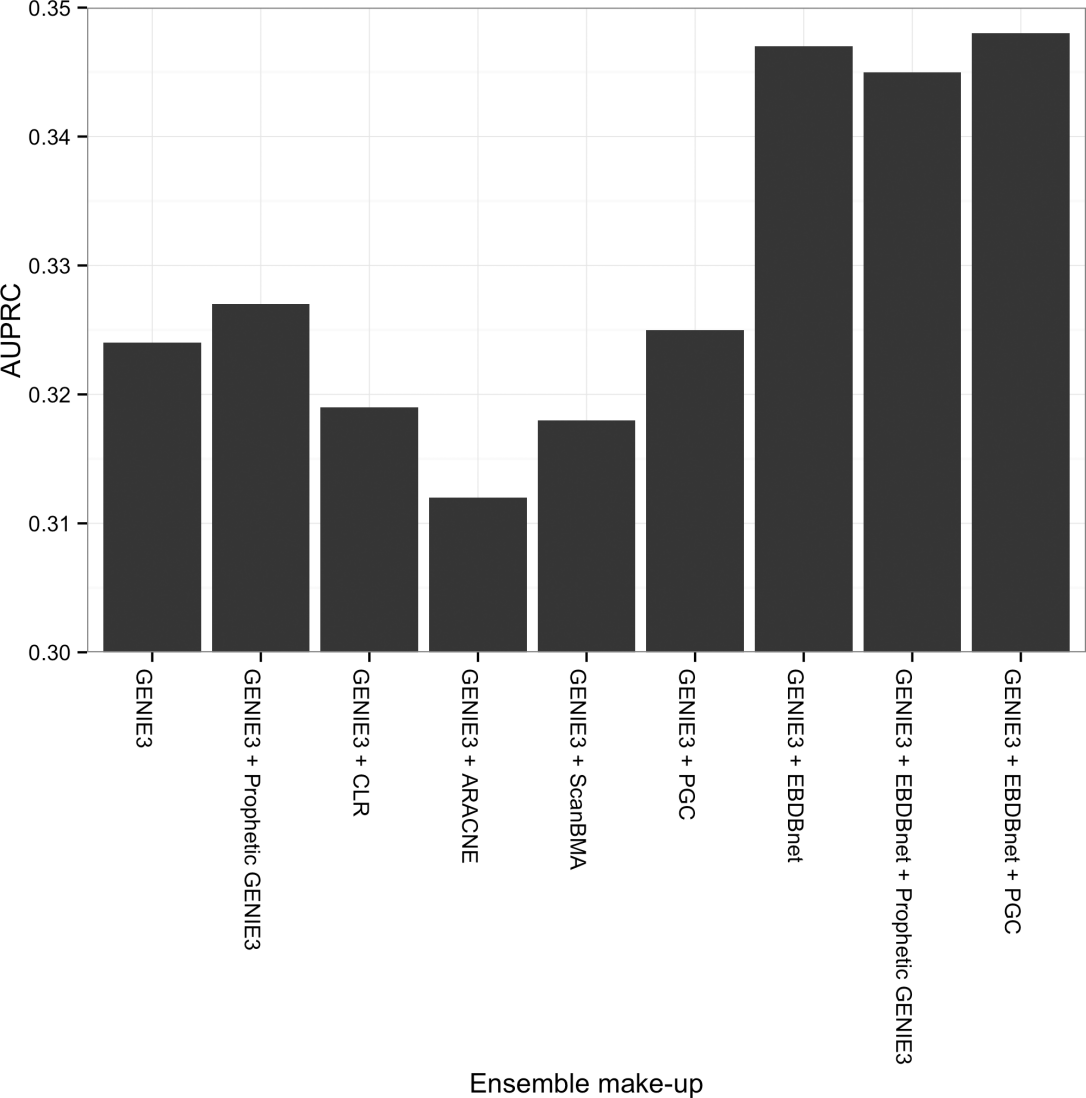
Tests on the Yeung dataset reveal PGC adds orthogonal information to improve performance of ensembles. Ensembles are constructed with methods added to the top performing method, GENIE3 (X-axis). Area under the Precision-Recall Curve (AUPRC) was used to measure performance (Y-axis). Only Prophetic GENIE3, Prophetic Granger Causality (PGC), and dynamic Bayes (EBDBnet) yielded additional performance improvement over the baseline GENIE3. The GENIE3, PGC and EBDBnet combination had the best performance.

To test the hypothesis that PGC provides weak, but complementary, network inferences when run on the Yeung dataset, we constructed ensembles by scaling each method’s output matrix to [0,1] (dividing by the largest value in the matrix) and taking the mean across all matrices to arrive at a single ensemble network. In order to explore the combinatorial space of possible ensembles, we used a forward selection method to construct ensembles. We started with the best performing single method, GENIE3, and added all other methods (Fig 3). Of this first round of ensembles, only Prophetic GENIE3, PGC, and EBDBnet improved on the results of GENIE3 and of these, EBDBnet provided the largest increase. Consistent with our hypothesis, PGC improved on GENIE3’s performance when added to it alone or when added to the GENIE3+EBDBnet combination.

### Biological implications of the PGC HPN network

**Fig 4:**
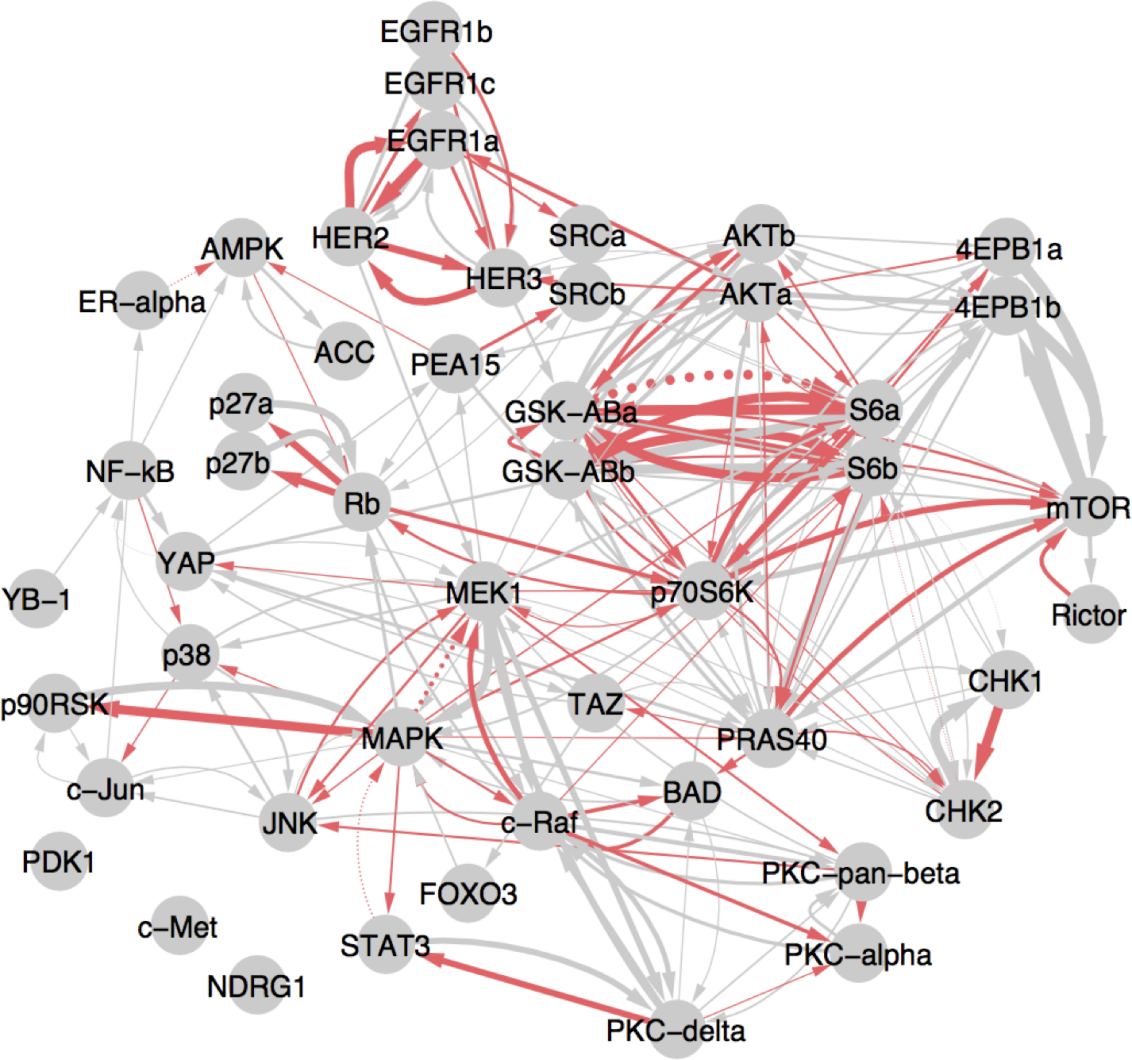
Cell-type vs. Stimulus ligand influence on the inferred HPN consensus network reveals a preponderance of cell-type interactions. Here we show the top 10 percent of interactions in the consensus network. ANOVA analysis on Granger coefficients was used to determine if interactions were cell-type dependent (red lines) or independent (grey) and if they were stimulus ligand-dependent (dotted) versus stimulus ligand-independent (solid). Line thickness reflects the inferred interaction strength. Cell-type-dependent interactions were much more common over stimulus ligand-dependent interactions suggesting that cellular context has an important influence on the underlying GRN. Proteins with more than one phosphorylation site are disambiguated with lower case letters following the protein name. Disambiguation of the identity of these probes appears in Supplemental S4 Table.

To investigate the properties and biological themes of the inferred PGC network for the HPN challenge, we selected the strongest top 10 percent (226) of the interactions in the consensus network for further analysis (see Fig 4). Mutual regulation and feedback was highly enriched in the consensus network: 176 of the consensus interactions had the reciprocal interaction also in the network (see S2 Table).

### Network interactions reflect cell type over stimulus ligand

We asked if the inferred networks are enriched more for cell line-dependent or stimulus ligand-dependent interactions. Differentiation confers cells with tissue-specific regulatory wiring. Transcriptional profiling by array platforms and RNA-sequencing have revealed that genome-wide gene expression follows a distinct tissue-specific pattern [41,42]. Cell of origin is readily classified from gene expression profiles using standard supervised learning methods, a trend that also holds for cell lines [43,44]. With few exceptions, the transcriptomes of primary tumors reflect the tissue from which they arise, suggesting that cell-of-origin still dominates tumor regulatory wiring rather than the numerous genomic perturbations [45]. Analyses using reverse-phase protein array data also confirm this observation. Thus, it seems plausible that the protein-level signaling networks inferred for the HPN cell lines might be cell-type dependent. In the case of the HPN challenge, all of the cell lines were derived from breast tumors. However, breast cancers are known to classify along several major subtypes, including the basal and luminal subtypes represented in the HPN dataset. Each subtype may represent a distinct cell-of-origin reflected in their highly pronounced transcriptional differences [46,47].

To test the cell-type dependency hypothesis, we considered the Granger coefficients as a function of the cell line and stimulus ligand condition under which it was derived and performed a two-way ANOVA to detect significant differences in interaction strength in the different conditions (S3 Table). We found 82 (33%) of the interactions to have cell-line-dependent Granger coefficients (p < 0.05). In contrast, only eight interactions (~3%) were found to be stimulus ligand-dependent. The cell type dependence of interactions can be observed in the differences between each cell line network, shown in S4–S7 Figs. As expected, this result supports the idea that cellular signaling networks inferred for a cell type under one state are likely to be applicable to another state for the same cell type.

In addition, several known subtype-dependent interactions were revealed from this analysis. For example, we observed a cluster of cell type dependent interactions involving the S6, p70S6K, GSK3B, and Akt proteins (Fig 4), which involve a set of cell proliferation-related genes that respond to nutrient signals such as the mTOR-AKT pathway. In support of these findings, p70 S6 kinase, which targets the ribosomal subunit S6 also in this subnetwork, has been found to act as an alternate route for downstream signaling when Akt is inhibited [48]. Thus, the cell-specific interactions in this subnetwork may reflect tissue-dependent growth-related signaling. Another strong cluster of cell type dependent interactions found by the method involve EGFR and HER2, which direct growth signaling in response to binding growth factors produced by the stromal environment. The EGFR-family protein, HER2, does not bind ligand on its own but instead modulates the activity of other EGFR-family members through heterodimerization. HER2 plays a well-documented role in aberrant growth signaling in breast and other cancers where HER2 gene copies are amplified and/or overexpressed leading to homodimerization and self-activation [49]. Therefore, the inference of a subtype-associated HER2-EGFR interaction reflects the observation that HER2 levels exert a strong regulatory influence on EGFR phosophoprotein levels in the HER2-amplified UACC812 cell line and only a small to moderate influence in the non HER2-amplified cell lines (MCF7, BT20 and BT549).

We also found a mutual regulation between p70S6K and the retinoblastoma protein (Rb) (see S8 Fig), representing a potentially novel cell-dependent interaction uncovered by PGC. Rb plays a critical role in regulating the entry into DNA synthesis during the cell cycle through interaction with chromatin modifying enzymes. Rb is downstream of estrogen receptor signaling, which is important in the ER-positive MCF7 cell line. The predicted mutual regulation of Rb and p70S6K is pronounced in MCF7, and may be an important previously uncharacterized source of crosstalk between ER signaling and canonical mTOR signaling.

### Novel interactions implicated in breast cancer signaling

PGC was able to reveal novel, previously undocumented (in the network prior) interactions beyond those already present in the network prior. Fifty-three of the 226 interactions in the consensus network were not in the top 10 percent of prior-supported interactions. These novel interactions (S8 Fig) are mutually distant in the network prior, but the time series data suggest causal relationships. While some links are likely false positives, others may suggest important new avenues of cancer research. For instance, the interaction between YAP and MEK1, previously undocumented in Pathway Commons [50] and not appearing in the prior, is suggested to have a role in liver cancer [51], in a study that was published concurrently with the DREAM8 contest. Rb and SRC were also detected to interact despite not being in the prior; some evidence for this interaction are also present in the literature [52].

Interestingly, YAP and NF-kB were implicated as mutually regulating in our analysis despite not having any previous support in the prior literature. These interactions would suggest a putative mechanism for linking the Hippo tumor suppressor pathway (of which Yap is a member [53]) to NF-kB-related apoptotic signaling.

### Genomic alterations underlie cell type specific wiring

We investigated the cell line dependency of the inferred network links from the HPN dataset. Specifically, we asked whether loss-of-function mutations influence the inferred regulatory networks. The unique combination of genomic lesions and mutations can result in major differences in the proteomes and their network wiring across subtypes as well as subtle differences within subtypes. The “natural” interaction neighborhood of a gene’s protein signaling network might be nearly or fully randomized in cell lines harboring loss-of-function mutations in the gene. In other words, a cell line with a loss-of-function mutation in gene X would be expected to have a different set of protein-protein signaling interactions involving the protein product of X compared to cell lines that have a wild-type copy of gene X. In this way, mutations could influence the disorder of a protein’s interactions and thereby help explain the cell type-dependent inferences. This type of “rewiring” due to mutations has previously been observed in a controlled setting [54].

To test the hypothesis that mutations influence cell type-dependent protein interactions, we retrieved all annotated coding mutations from the Cancer Cell Line Encyclopedia that occur in the cell lines and proteins queried by the DREAM8 challenge. Six mutations occur in the proteins and cell lines of interest (see Table 1). We looked at the upstream and downstream interactions involving the mutated proteins to determine if these interactions were noticeably perturbed in the cell lines where the mutation occurred. To measure this, we compared the normalized PGC coefficients |C|_*,*_/ max (|C|_*,*_) on interactions involving genes mutated in a cell line to coefficients computed for cell lines in which the gene is not mutated.

**Table 1:** Impact of mutations on local network.

Mutation	Cell line	Mutation type	Downstream interaction Wilcoxon p-value	Upstream interaction Wilcoxon p-value
CHEK2	UACC812	nonsense	**0.011**	0.19
MAPK8	UACC812	missense	**0.032**	**0.00033**
MET	MCF7	intron deletion	0.67	**0.03**
PRKCQ	UACC812	missense	**0.0009**	0.23
RB1	BT20	missense	0.29	0.092
RPS6KB1	BT20	3' UTR insertion	**0.0018**	**0.032**

Wilcoxon tests were used to determine if the weight of the interactions involving a particular gene have decreased in cell lines for which the gene is mutated compared to those cell lines in which it is not. Mutations with significant (p<0.05) loss of interaction strength appear in bold.

**Fig 5:**
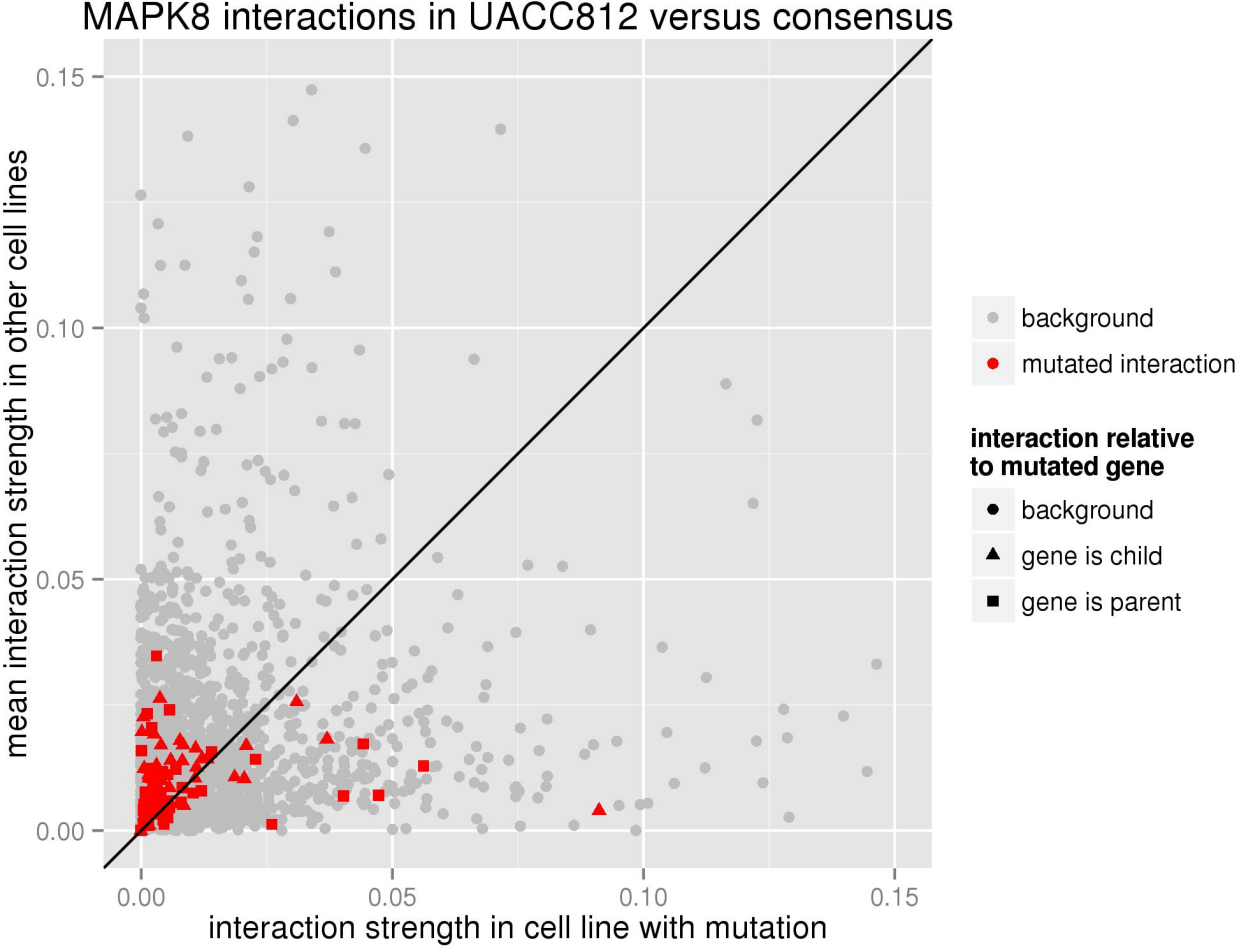
Evidence of mutational disruption network activity of MAPK8. Interaction strengths involving JUN N-terminal Kinase (MAPK8) in the mutant UACC812 cell line are lower than in wild type cell lines. Interaction strengths were calculated as the normalized Granger coefficients derived in each cellular context. Each point is an interaction and points that appear above the line of equality (*Y* = *X*) indicate loss of function. Interaction strengths derived from all other interactions not involving MAPK8 are shown as the background (grey dots). Both the upstream and downstream interactions of MAPK8 (red) are significantly disrupted.

We found that five out of six mutated genes had detectable decreases in interaction coefficients, either downstream or upstream of the gene, compared to the wild-type cell lines. Four out of six of the mutated genes had significant decreases in downstream interactions (P<0.05; Wilcoxon non-parametric test), indicating a loss-of-function of these genes. Three out of the six genes had significant decreases in the Granger coefficients associated with their upstream interactions, which may represent a decrease in their coherent regulation, phosphorylation or detection. The strongest disruption of gene function occurred in MAPK8 in the UACC812 cell line (Fig 5).

## Discussion

A number of methods have been published for inferring gene regulatory networks from time series data since the end of the DREAM contest. For instance Zhou et al. 2015 [55] have utilized an ensemble resampling method to increase robustness of their method over competing methods. Liu et al. (2015) [56] take a Bayesian approach to add and prune edges to maximize the posterior likelihood of the data. Agdham et al. (2015) [57] attacked the inference problem with an information-theoretic approach, which performs well in their benchmark but cannot infer edge directionality. Zhang et al. (2014) [58] used a conditional mutual information measure to prune down from a fully connected graph by eliminating nodes with mutual information explained by intermediaries very much like the previously published ARACNe method [36]. Nair et al. (2015) [59] combine a Bayes net framework with additional topological node degree constraints that mimic observed biological networks, thereby reducing the complexity of the search space. All of these approaches bring a new aspect to regulatory network inference. However, considering information from future time points at each regression point remains novel and, as PGC and Prophetic GENEI3 here reveal, should be considered when approaching future network inference challenges.

We have adapted Granger’s Nobel Prize winning work on inferring economic relations in time series data to predicting causal protein interactions. The Prophetic Granger Causality (PGC) method was a top-performer in the DREAM8 competition, producing interactions with a higher likelihood of representing causal connections compared to other methods. DREAM8 evaluated methods using wet lab experiments conducted after all algorithm predictions were collected. Thus, the results of the challenge provide compelling evidence that the Granger approach is worth considering for causal inference problems of the sort presented by the challenge. While any particular challenge has a certain element of randomness in the methods that it nominates, the fact that the Prophetic Granger method outperformed 73 other submissions in the final scoring round lends credibility to its strength. The goal of any DREAM challenge is to provide an unbiased platform for method comparison. Bootstrap samples of the data are used by the organizers to ensure that the final rankings are robust. Here, we attempted to characterize the ability of PGC to generalize to a new yeast dataset and found that, while a weak predictor on its own, it significantly improves the performance of ensembles, likely through the contribution of independent predictive power.

Granger Causality has been applied to systems biology in the past [27]. Indeed, upon conclusion of the HPN 1A sub-challenge, a closely related approach was published [60]. However, the non-uniform temporal intervals of the DREAM8 data prevent the straightforward application of the method; the Granger approach is typically used when the time series is made up of regularly spaced intervals, leading to all time points in the series contributing to the same regression model. In the DREAM8 case, the observations cannot be viewed as states in a discrete Markov process since the interval between time points **t** and (**t** + 1) could be different from the interval between (**t** + 1) and (**t** + 2).

As we demonstrate with the augmented GENIE3 approach, the use of both past and future time points in deriving causal links can be extended to other methods, such as non-linear regression or mutual information networks. The prophetic augmentation may benefit from picking up subtle dependencies detected by reverse regression that are missed by forward regression. Regressing in the usual forward direction, in which the target is the response variable, may miss a connection between an upstream regulator and one of its targets because the influence from the regulator may be subtle (e.g. below some noise threshold), or it may be redundant when possibly other regulators are considered. However, regressing in the reverse direction, when the regulator is used as the response variable, provides a second opportunity to detect the link because the target’s data may be partially predictive of the regulator’s past state.

The prophetic extension also has the advantage of using all of the data for each link prediction task to boost statistical power, regardless of what method is used. The modest gain in performance obtained by averaging the PGC solutions, along with the good performance of the Prophetic GENIE3 approach (which used all of the data simultaneously) suggests that the use of more data outweighs the importance of describing cell line-dependent differences in the networks. In addition, the prior helped to cut down on the apparent false positives resulting from the regression step, which can be viewed as another example in which the incorporation of background knowledge is useful for tasks in which limited training data is provided.

In deriving the PGC solution to the HPN-DREAM challenge, there are two aspects that were ignored: 1) the sign of influence defining if an interaction is activating or repressive and 2) the length of time between predictors and response variables. In such cases where different time scales are queried by an experiment, as was true for the DREAM8 challenge, one could obtain prediction rules associated with both fast and slow acting mechanisms. For example, interaction “speed” could be estimated with Granger coefficients associated with each interval to form a weighted average of interaction time. While the HPN challenge data may be underpowered for this analysis, other datasets with more proteins and observations might uncover biological underpinnings and gene functions correlated to such estimated interaction timescales.

The PGC solution to the HPN challenge provides several new biological insights. In addition to the interactions identified mTOR-AKT pathway genes and the EGFR family, several novel interactions such as MEK1-YAP and RB1-p70S6K were uncovered. PGC provides context-dependent information about under what cell types and perturbations interactions could operate.

We find a propensity of cell type- over stimulus ligand-dependent protein-protein interactions among the inferred links. If this trend generally holds it would suggest pooling together datasets to construct cell-specific protein networks to use as a backdrop for further fine-tuned modeling of particular perturbations. Interestingly, we were able to show a quantifiable change in the protein interaction circuitry as a function of a cell’s genetic background. Mutations of a gene in a cell line disrupt the circuitry of the network neighbors of the gene’s protein product. Thus, the cell type specificity of signaling networks can be explained at least in part by the hard-coded alterations in a cell’s genome. As new epigenetic data become available, such as those from the Epigenetic Roadmap [61], network reconstruction methods will be able to leverage a rich set of information to create accurate cellular models across tissues and developmental stages.

## Supporting information

S1 File Information describing how the network prior was calculated for the main analysis. (DOCX)

S1 Table Performance on other datasets- area under the precision-recall and receiver-operator curves for the in silico DREAM 4 data and the Yeung yeast regulation dataset. Temporality Considered shows this methods use which time points in attempting to determine causality; “all” means all time points, before, during and after the time point being considered, “current” means only the present time point, and “t-1” means only the immediately previous time point.
(XLSX)

S2 Table Phosphosites with inferred mutual regulation- these probes demonstrated mutual causality, increasing belief that they are involved in common functional modules. (XLSX)

S3 Table Edge weights are reported for the consensus, cell type-specific, prior alone, and consensus Prophetic Granger Solutions. We also include the Prophetic GENIE3 results here, since this algorithm performed best in the post-contest analysis. We indicate for each edge whether it was found to depend on the cell type or the stimulus ligand condition by an ANOVA test (p<0.05).
(XLSX)

S4 Table Disambiguation for phosphosites on the same gene. Six genes had multiple phosphosites, each one indicated with a different lowercase letter after the protein name. Letters indicate the particular phosphosite of a protein.
(XLSX)

S1 Fig HPN DREAM8 1A performance of PGC mixed with the heat-diffusion prior. Contribution of the prior increases to the right. Error bars correspond to the standard error of the mean produced by subsampling the test data 100 times. The grey horizontal lines correspond to the top 2 entries in the contest; the winning entry, which was a 50–50 mix of the heat diffusion prior and the PGC solution, and the second best entry, which was the prior alone. The best performing mix was the 80/20 prior to PGC ratio, which achieved an average AUROC of 0.797.
(PDF)

S2 Fig The performance of the heat diffusion prior alone as a function of the diffusion time parameter *d*. Mean AUC is the mean area under the receiver-operator curve used for evaluation in the HPN DREAM Challenge 1A.
(PDF)

S3 Fig The top 10 percent of edges obtained from the heat diffusion prior. (PDF)

S4 Fig Performance of various methods on the HPN DREAM8 challenge 1A after combination with the prior. All combinations were done in the same manner as PGC; each was divided by the largest entry so that the scaling existed on [0,1], then averaged with the prior. Team Names appear in parentheses. See ^2^ for method details.
(PDF)

S5 Fig The top 10 percent of all interactions detected in the UACC812 cell line after combining with the prior. (PDF)

S6 Fig The top 10 percent of all interactions detected in the MCF7 cell line after combining with the prior. (PDF)

S7 Fig The top 10 percent of all interactions detected in the BT549 cell line after combining with the prior. (PDF)

S8 Fig The top 10 percent of all interactions detected in the BT20 cell line after combining with the prior. (PDF)

S9 Fig The top 10 percent of consensus interactions that were not also in the top 10 percent of prior interactions. These interactions suggest novel (or undocumented by Pathway Commons) biology. Red interactions are cell-line dependent.
(PDF)

S10 Fig Illustration of how a regulatory interaction from a regulator R to a target T, which is undetected using forward regression can be detected using the reverse analysis. (Top) Forward regression, where T is the response, misses the link R->T due to presence of other regulators R1, R2, and R3 that explain target T’s state sufficiently when used as predictor variables (i.e. R’s information is redundant as a predictor given the other regulators). (Bottom) Reverse direction, where R is the response, detects the R->T link since T provides some partial explanatory power as a predictor of R’s state in the past.
(PDF)
