# Supplementary material for: Prophetic Granger Causality to infer gene regulatory networks
Source: PLoS One. 2017 Dec 6;12(12):e0170340. doi: 10.1371/journal.pone.0170340 (PMC5718405; doi:10.1371/journal.pone.0170340)
Supplement: S1 File — (DOCX) [file pone.0170340.s001.docx]

**SUPPORTING INFORMATION**

For: “Prophetic Granger Causality to infer Gene Regulatory Networks”

by Daniel E. Carlin, Evan O. Paull, Kiley Graim, Chris Wong, Adrian Bivol, Peter Ryabinin, Kyle Ellrott, Joshua M. Stuart, and Artem Sokolov

**SUPPLEMENTAL METHODS**

**A. Derivation of the network prior**

We seek a reasonable starting point for network reconstruction, one that uses links between proteins based on what is known in the literature. Such a *network prior* might aid in the interpretation of a dataset like the phosphoproteomics data if enough of the protein-protein interactions are relevant to the condition(s) represented in a study. For the purposes of this challenge we used established pathway collections (gathered from Pathway Commons [[1]](https://paperpile.com/c/9XrwYP/7j1vj)), since their protein interactions tend to fit most closely with a notion of causal influence. We assume that the transcriptional and post-transcriptional interactions declared in these pathway databases imply that a perturbation to an upstream regulatory protein will lead to either direct or indirect perturbation of a downstream target protein that is connected to the regulator via a directed path. In biological data, feedback mechanisms may be at work, such that perturbations in a downstream protein lead to perturbations in the upstream regulators. Thus, for the prior, we infer an undirected Gene Interaction Network (GIN) and let the ambiguity of the directionality between cause and effect be resolved in the later PGC regression step.

In general, we are given a set of proteins of interest *P* for which we have temporal observations, such as the HPN phosphoproteomics data. *P* may be a small set of proteins scattered over a pathway collection, such that there are no direct links between them. To resolve this, we need a measure of proximity between each pair of proteins in *P*. We chose heat diffusion, which has been shown to be a useful proxy for functional relatedness, especially to identify sub-networks highly mutated in cancer [[2–5]](https://paperpile.com/c/9XrwYP/PVPqg+qqEsi+891rc+Oeyn8). It is also computationally efficient to calculate, even on large graphs, and has more granularity and robustness to the presence of errors in graph connectivity than shortest-path approaches. In addition, heat diffusion has been shown to provide a better measure of causal influence between proteins than network flow and shortest path algorithms [[5]](https://paperpile.com/c/9XrwYP/Oeyn8).

Our approach was to first create a GIN by merging all pathways together and then calculate heat diffusion on the merged network to obtain the network prior. We merge all pathway networks in Pathway Commons that contained at least two of the proteins in *P* to form a global GIN. To do this, each pathway is first reduced to an undirected graph where each node corresponds to a protein and each edge to an interaction. For proteins that act in a complex, each of the complex constituents is assigned an edge to the targets of the complex. The merge operation is performed by taking the union of all links found in the set of separate input pathways. The resulting GIN is undirected, describing general relations between genes and/or their gene products.

Once the global GIN is obtained, the network proximity between two distinct proteins *i* and *j* in *P* was measured by treating protein *i* as a heat source and recording the diffused heat at protein *j* as *H(i,j)*. Note that this metric is symmetric: *H(i,j)=H(j,i).* The heat diffusion process was run as described by Vandin *et al.* [[3]](https://paperpile.com/c/9XrwYP/qqEsi). Briefly, if *L_p_* is the graph Laplacian of the global GIN and *d* is the diffusion time parameter, then the heat kernel is calculated via *H = expm(-L_p_*d)*, where *expm()* is the matrix exponential. For the HPN challenge submission, we fixed *d* at 0.1, as suggested by Vandin, *et al.* [[3]](https://paperpile.com/c/9XrwYP/qqEsi). The effect of changing *d* on the performance is explored in S1 Fig. To obtain the final network prior, we set the diagonal entries *H(j,j)* to zero to preclude self links. Finally, all probes assigned to the same protein were assumed to have the same biological prior information.

Importantly, the calculation of the heat kernel uses no information about the training data other than the proteins of interest, P, and could be computed once for the global GIN and reused as the prior for all experiments.

We derived a network prior from the Pathway Commons database version 3

^3^. We found 263 pathways that contain two or more of the proteins with antibodies measured in the HPN challenge. These pathways were used with heat diffusion to infer protein-protein interactions for the network prior (see Methods). This heat diffusion process requires a single “diffusion time”^7^ parameter to be set, which we found to be optimal at 0.13 for the HPN dataset (S2 Fig). However, we found the performance to be robust for any value above 0.08, including the value used for the official HPN submission (0.10), which is in good agreement with prior literature [[3]](https://paperpile.com/c/9XrwYP/qqEsi). Notably, when the interactions from the network prior were submitted as the inferred HPN network, on their own they achieved the second highest score in the DREAM8 sub-challenge 1A. Thus, the summary knowledge contained in the network prior has many relevant causal paths between the proteins assayed in the DREAM8 dataset. This provides confidence, at least in the case of the HPN challenge, to initialize network inference with a collection of dataset-independent interactions and then refine the predictions based on the correlations detected in the time series data.

The resulting prior included all but four of the query proteins (NDRG1, PDK1, PEA15, and YBX1). For these genes the prior weights of all connecting edges is set to zero. To visualize these interactions, the strongest 10% of links in the prior network were extracted to inspect the biological relevance of the inferences (S3 Fig).

**B. Difference between the network priors used in this paper and the HPN DREAM Challenge**

Note that the solution submitted for the HPN challenge used a slightly different network prior formulation than what is described here. At the time of the challenge, the heat kernel was computed on each pathway separately and then combined in a greedy fashion where each pathway kernel replaced the entries corresponding to its protein interactions in the B matrix in the order which the pathway kernels were computed. After the completion of the challenge and during the post-contest analysis, we found that computing the heat kernel on the combined graph of all pathways achieved slightly better performance (0.783 average AUC compared to 0.771 of the submitted prior) and also was pathway order independent. This also accounts for the different scores for the overall PGC approach appearing here (0.785) and on the contest leaderboard (0.782).

**SUPPLEMENTAL REFERENCES**

1. [Cerami EG, Gross BE, Demir E, Rodchenkov I, Babur O, Anwar N, et al. Pathway Commons, a web resource for biological pathway data. Nucleic Acids Res. 2011;39: D685–90.](http://paperpile.com/b/9XrwYP/7j1vj)

2. [Qi Y, Suhail Y, Lin Y-Y, Boeke JD, Bader JS. Finding friends and enemies in an enemies-only network: a graph diffusion kernel for predicting novel genetic interactions and co-complex membership from yeast genetic interactions. Genome Res. 2008;18: 1991–2004.](http://paperpile.com/b/9XrwYP/PVPqg)

3. [Vandin F, Upfal E, Raphael BJ. Algorithms for detecting significantly mutated pathways in cancer. J Comput Biol. 2011;18: 507–522.](http://paperpile.com/b/9XrwYP/qqEsi)

4. [Hofree M, Shen JP, Carter H, Gross A, Ideker T. Network-based stratification of tumor mutations. Nat Methods. 2013;10: 1108–1115.](http://paperpile.com/b/9XrwYP/891rc)

5. [Paull EO, Carlin DE, Niepel M, Sorger PK, Haussler D, Stuart JM. Discovering causal pathways linking genomic events to transcriptional states using Tied Diffusion Through Interacting Events (TieDIE). Bioinformatics. 2013;29: 2757–2764.](http://paperpile.com/b/9XrwYP/Oeyn8)

6. [Huynh-Thu VA, Irrthum A, Wehenkel L, Geurts P. Inferring regulatory networks from expression data using tree-based methods. PLoS One. 2010;5. doi:](http://paperpile.com/b/9XrwYP/ddRT)[10.1371/journal.pone.0012776](http://dx.doi.org/10.1371/journal.pone.0012776)
