# Supplementary figures and images for: Prophetic Granger Causality to infer gene regulatory networks
Source: PLoS One. 2017 Dec 6;12(12):e0170340. doi: 10.1371/journal.pone.0170340 (PMC5718405; doi:10.1371/journal.pone.0170340)

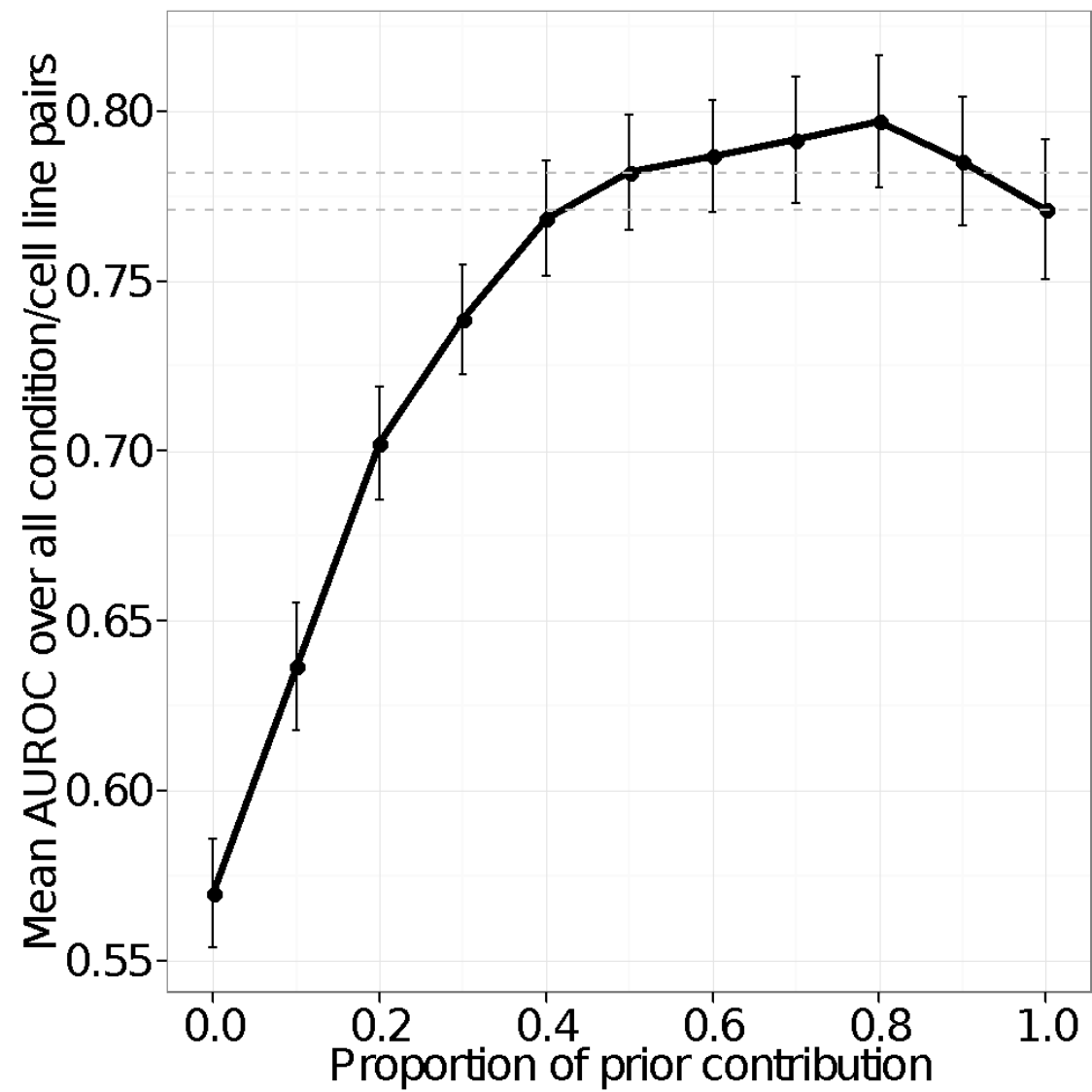

Supplement: S1 Fig — Contribution of the prior increases to the right. Error bars correspond to the standard error of the mean produced by subsampling the test data 100 times. The grey horizontal lines correspond to the top 2 entries in the contest; the winning entry, which was a 50–50 mix of the heat diffusion prior and the PGC solution, and the second best entry, which was the prior alone. The best performing mix was the 80/20 prior to PGC ratio, which achieved an average AUROC of 0.797. (PDF) [file pone.0170340.s006.pdf]

# Prior performance with varying diffusion time parameter

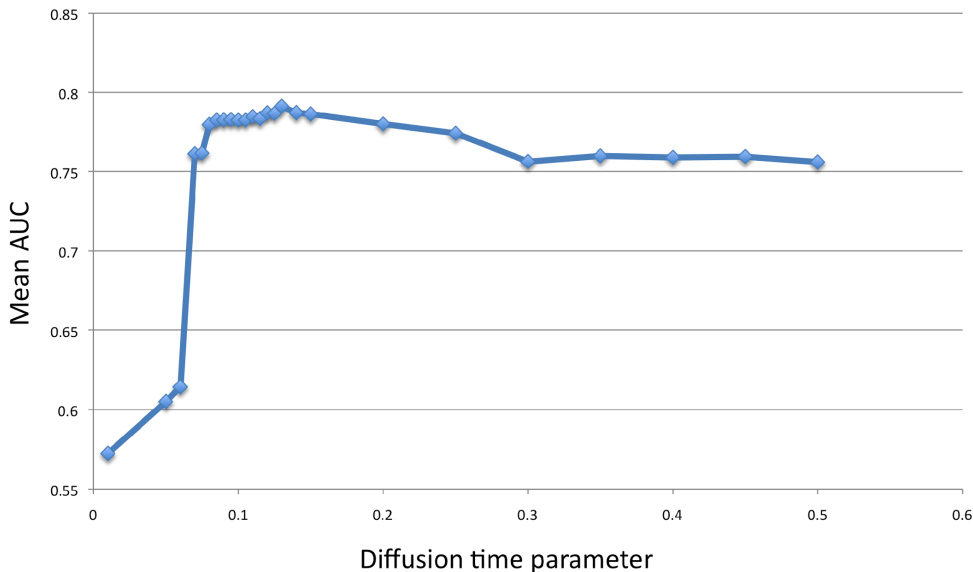

Supplement: S2 Fig — Mean AUC is the mean area under the receiver-operator curve used for evaluation in the HPN DREAM Challenge 1A. (PDF) [file pone.0170340.s007.pdf]

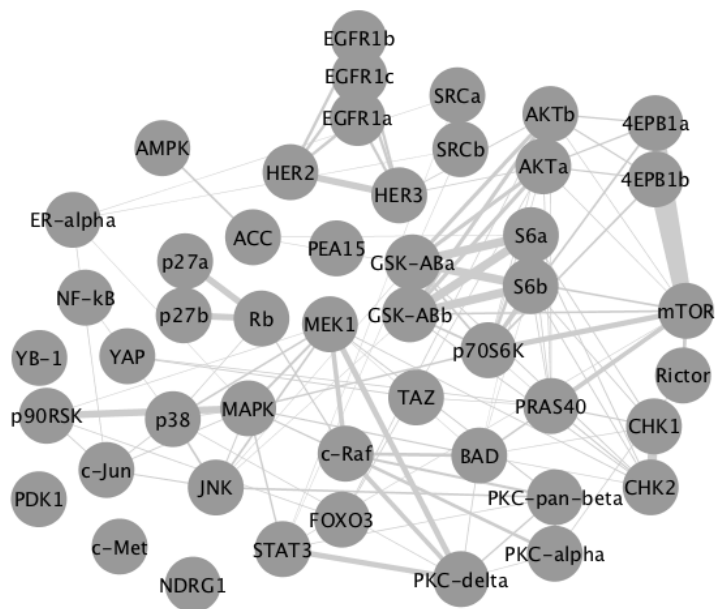

Supplement: S3 Fig — (PDF) [file pone.0170340.s008.pdf]

**Performance of various methods after averaging with prior**

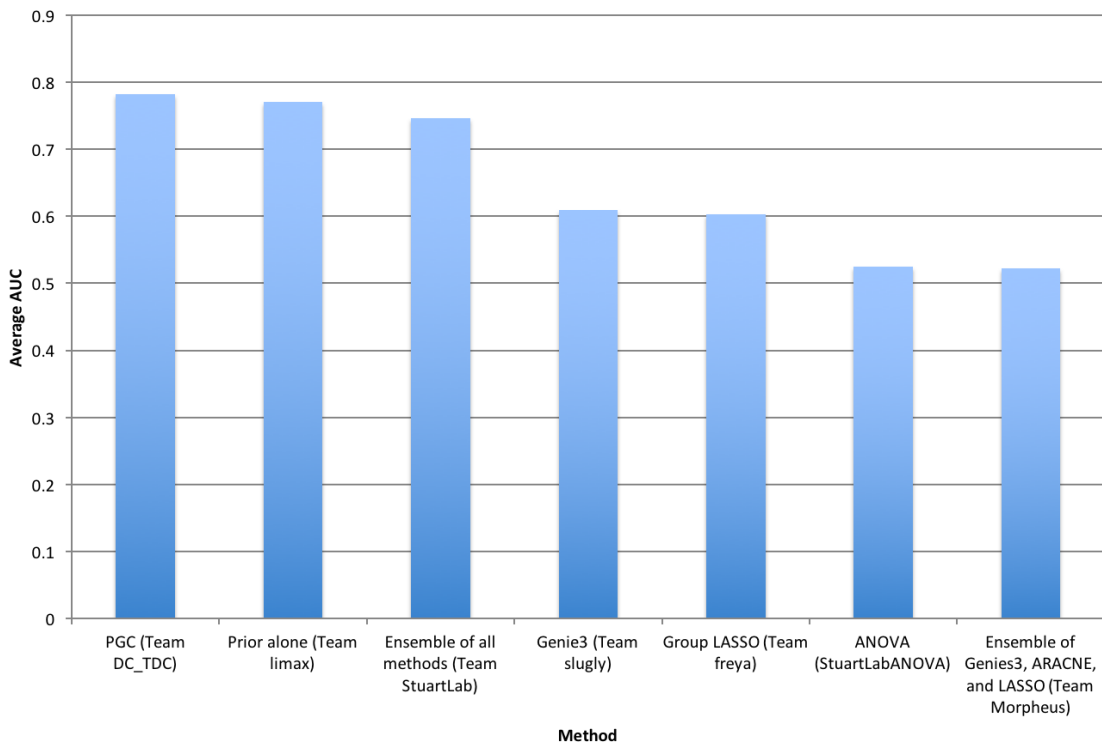

Supplement: S4 Fig — All combinations were done in the same manner as PGC; each was divided by the largest entry so that the scaling existed on [0,1], then averaged with the prior. Team Names appear in parentheses. See 2 for method details. (PDF) [file pone.0170340.s009.pdf]

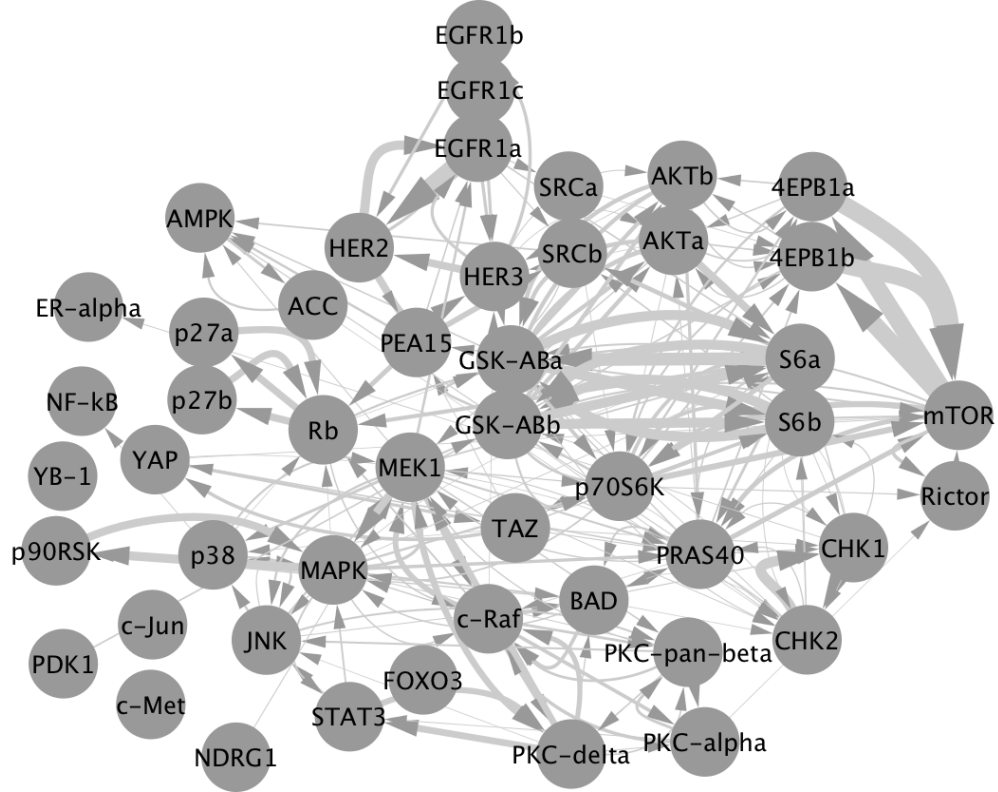

Supplement: S5 Fig — (PDF) [file pone.0170340.s010.pdf]

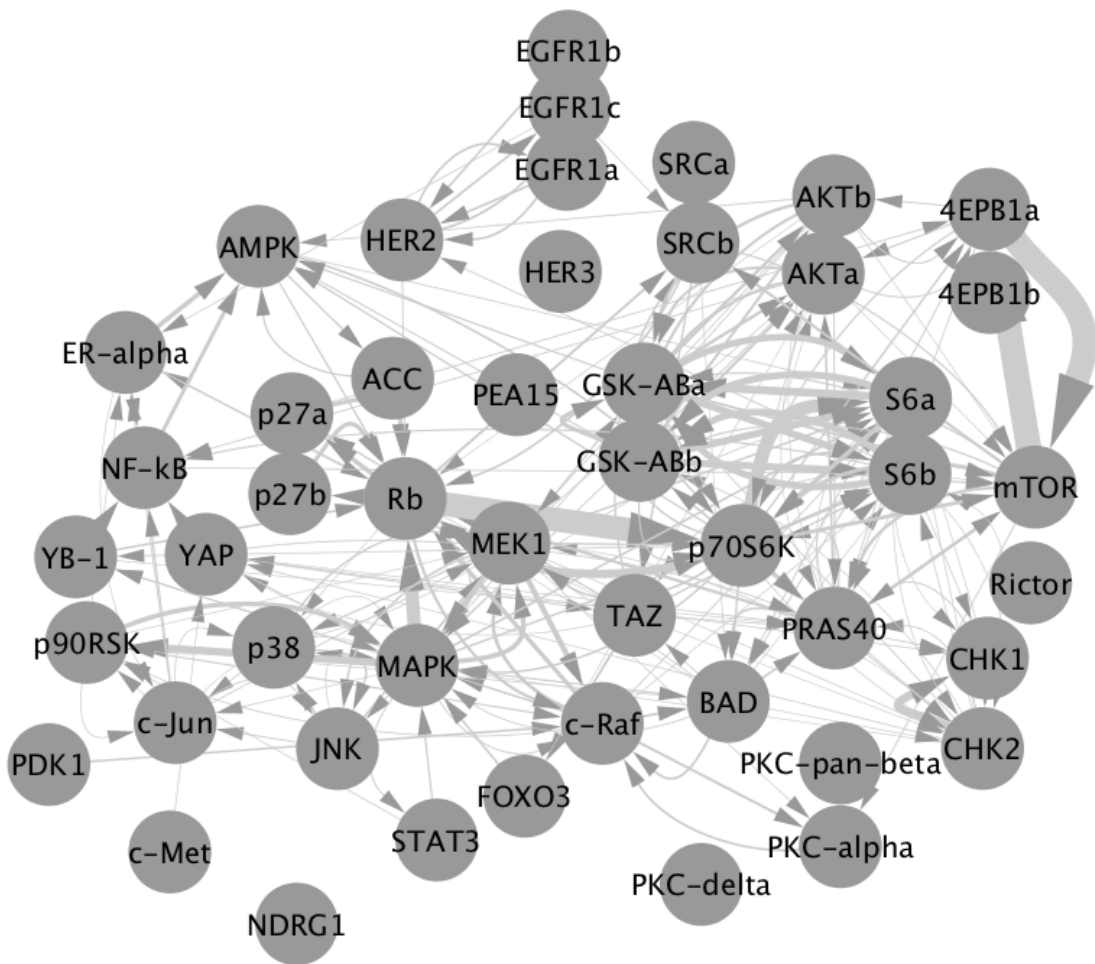

Supplement: S6 Fig — (PDF) [file pone.0170340.s011.pdf]

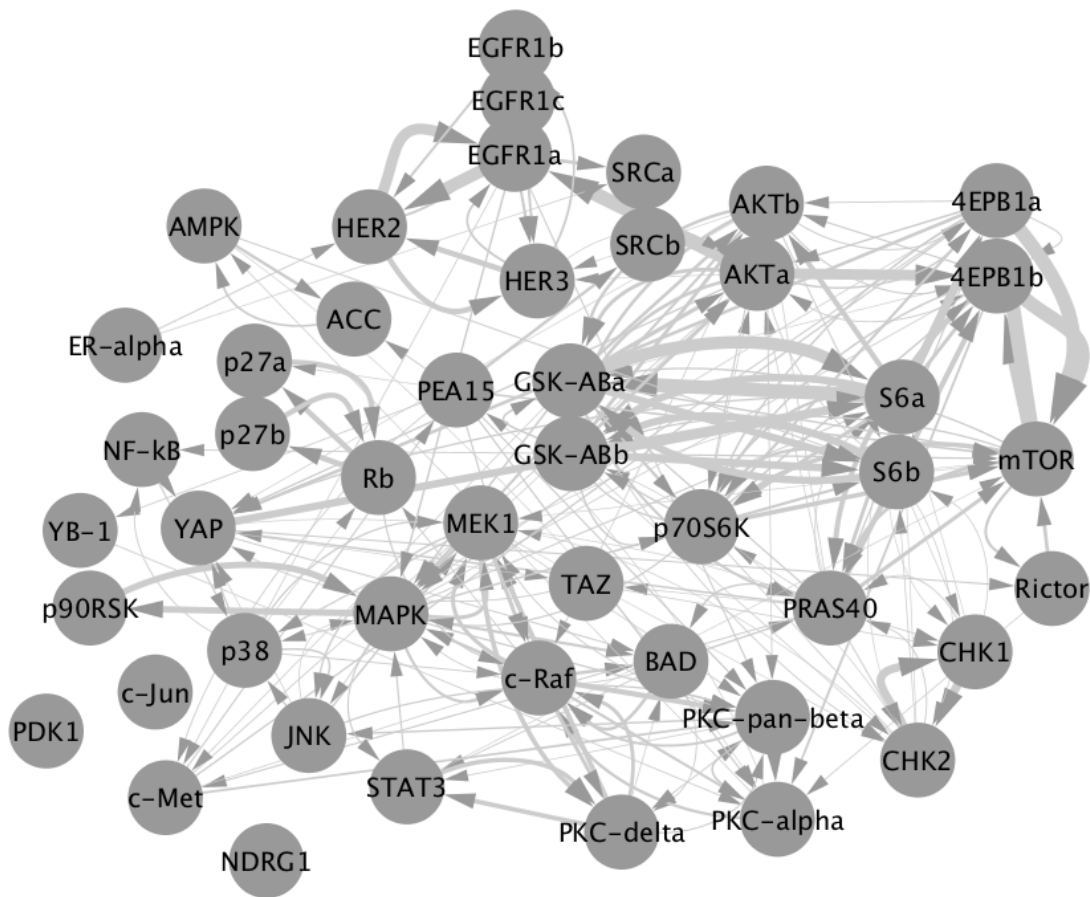

Supplement: S7 Fig — (PDF) [file pone.0170340.s012.pdf]

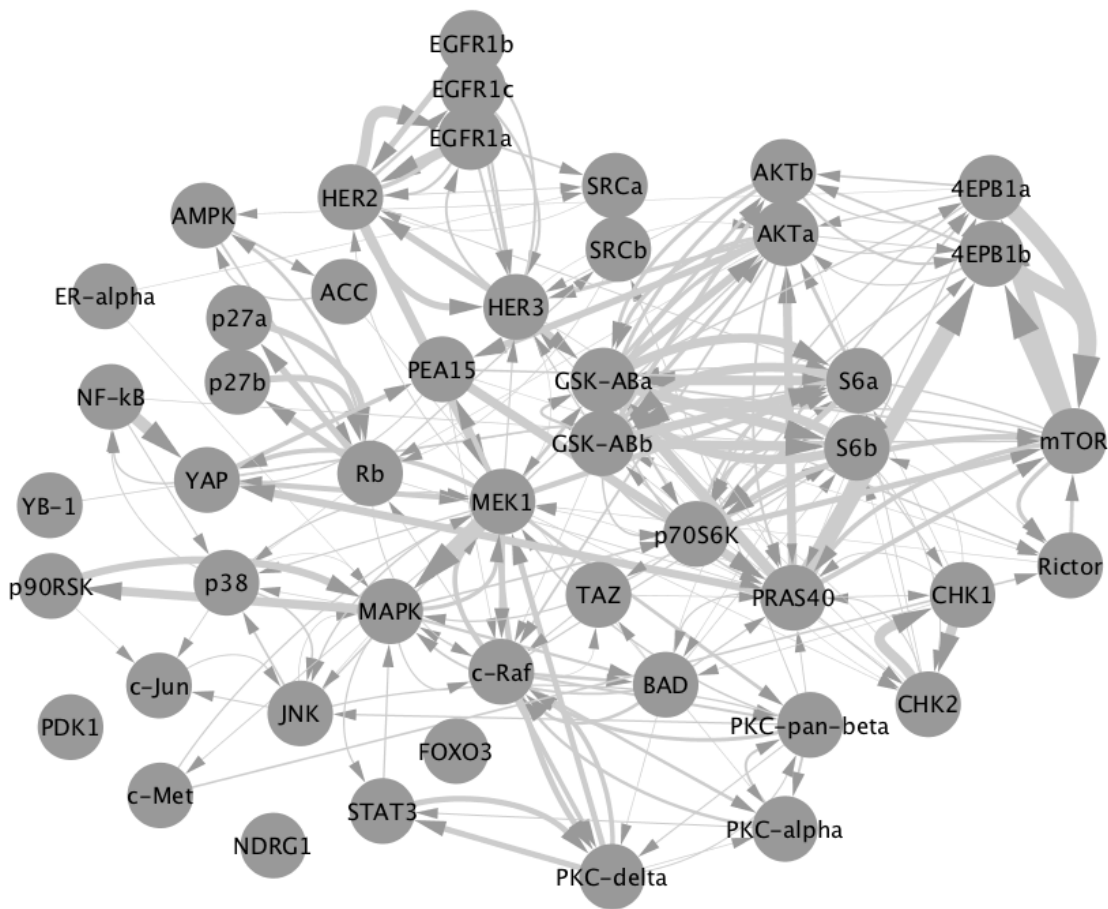

Supplement: S8 Fig — (PDF) [file pone.0170340.s013.pdf]

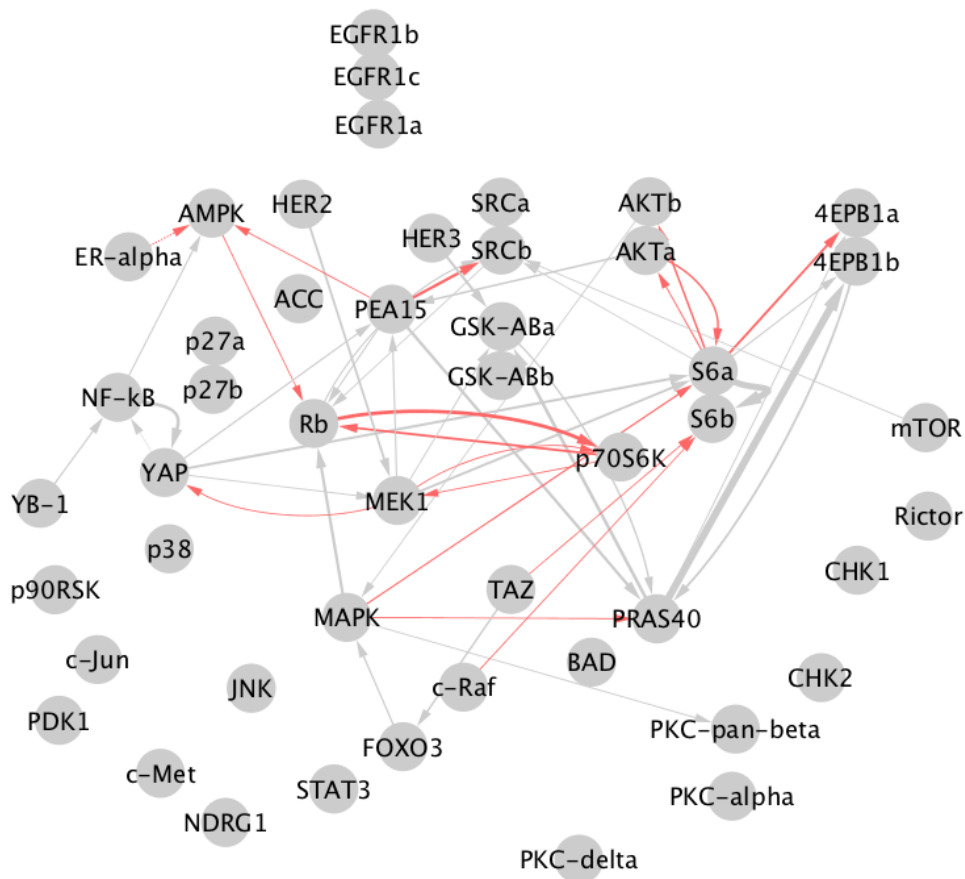

Supplement: S9 Fig — These interactions suggest novel (or undocumented by Pathway Commons) biology. Red interactions are cell-line dependent. (PDF) [file pone.0170340.s014.pdf]

Forward  
Regression

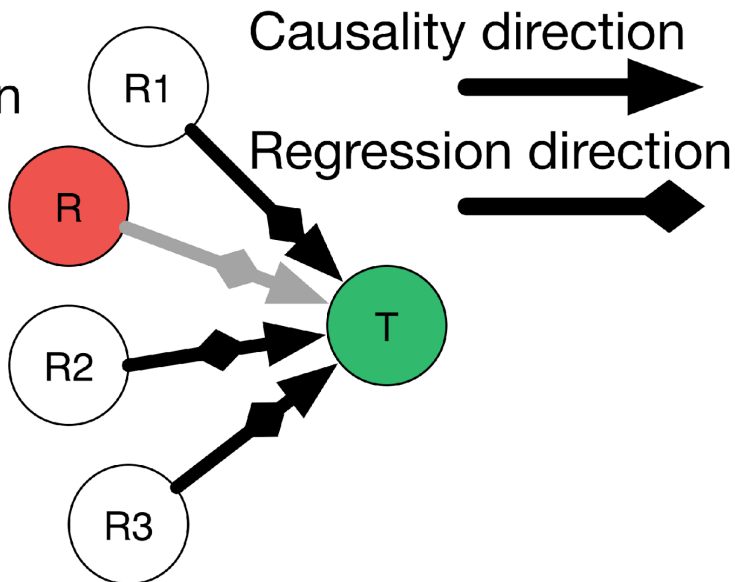

Reverse  
Regression

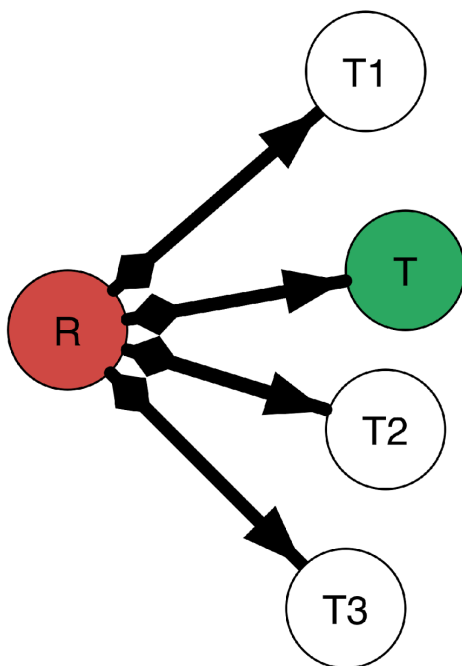

Supplement: S10 Fig — (Top) Forward regression, where T is the response, misses the link R->T due to presence of other regulators R1, R2, and R3 that explain target T’s state sufficiently when used as predictor variables (i.e. R’s information is redundant as a predictor given the other regulators). (Bottom) Reverse direction, where R is the response, detects the R->T link since T provides some partial explanatory power as a predictor of R’s state in the past. (PDF) [file pone.0170340.s015.pdf]
